# Aggressiveness Overcomes Body-Size Effects in Fights Staged between Invasive and Native Fish Species with Overlapping Niches

**DOI:** 10.1371/journal.pone.0029746

**Published:** 2012-01-17

**Authors:** Fábio Henrique Carretero Sanches, Caio Akira Miyai, Tânia Márcia Costa, Ronaldo Adriano Christofoletti, Gilson Luiz Volpato, Rodrigo Egydio Barreto

**Affiliations:** 1 Departamento de Fisiologia, Instituto de Biociências, Caunesp, UNESP, Botucatu, São Paulo, Brazil; 2 Campus Experimental do Litoral Paulista, UNESP, São Vicente, São Paulo, Brazil; 3 Universidade de São Paulo, Centro de Biologia Marinha (CEBIMar/USP), São Sebastião, São Paulo, Brazil; University of Plymouth, United Kingdom

## Abstract

Approximately 50 years ago, Nile tilapia were accidentally introduced to Brazil, and the decline of pearl cichlid populations, which has been intensified by habitat degradation, in some locations has been associated with the presence of Nile tilapia. There is, however, little strong empirical evidence for the negative interaction of non-native fish populations with native fish populations; such evidence would indicate a potential behavioural mechanism that could cause the population of the native fish to decline. In this study, we show that in fights staged between pairs of Nile tilapia and pearl cichlids of differing body size, the Nile tilapia were more aggressive than the pearl cichlid. Because this effect prevailed over body-size effects, the pearl cichlids were at a disadvantage. The niche overlap between the Nile tilapia and the pearl cichlid in nature, and the competitive advantage shown by the Nile tilapia in this study potentially represent one of several possible results of the negative interactions imposed by an invasive species. These negative effects may reduce population viability of the native species and cause competitive exclusion.

## Introduction

Invasive species are a serious ecological problem. The management of non-native populations that become naturalised requires attention because intense competition for limited resources usually occurs between native and invader species [1] and can alter natural communities [2]. Niche overlap is usually high in this situation, and intense competition may cause the rapid decline and even the extinction of native populations [1], [3]–[7]. This effect has become widespread in aquatic ecosystems, where the introduction of exotic fish species has caused deleterious consequences to native fish species worldwide [8]–[12]. These negative effects on native species have been attributed to the higher competitive ability of the invasive fish species [10], [12]. However, strong empirical evidence of negative interactions between non-native fish populations and native fish populations is lacking. Such evidence should demonstrate the mechanisms underlying the invader's competitive advantage over the native fish and should, therefore, potentially suggest approaches to the management of the invasive species.

The Nile tilapia was accidentally introduced in Brazil approximately 50 years ago. This cichlid is one of the most frequently cultivated fish worldwide and is of great economic importance, particularly for Brazilian aquaculture [13]. However, the Nile tilapia is a generalist species, and its ability to adjust readily to stream environments probably facilitated its rapid spread throughout the Brazilian river systems [14]. The decline of the pearl cichlid populations has been associated with the occurrence of Nile tilapia, and this decline has been further intensified by habitat degradation [14]. In fact, other species are reported to be extinct in other localities worldwide where the Nile tilapia has been introduced [8], [15]. In laboratory studies, the displacement of a native species (sunfish, *Lepomis miniatus*) from their preferred structured habitats has been linked to agonistic interactions with Nile tilapia [12]. The effect of the Nile tilapia on pearl cichlid populations is not the only deleterious effect of this exotic species. The pearl cichlid is a predator of the snail that is the vector for *Schistosoma* [16], whereas the Nile tilapia does not feed on molluscs [17]. The loss of this predatory species could therefore cause problems for human health.

The Nile tilapia and the pearl cichlid are both aggressive fish species [18]–[21], yet the Nile tilapia appears to show a competitive advantage over the pearl cichlid [14]. In nature, the most common type of resource competition involves aggressive disputes [22], and the contestants are likely to differ in their fighting ability, termed ‘resource-holding potential’ (RHP) [23]–[25], reviewed by Arnott & Elwood [26]. Body size is commonly used as a proxy for RHP and is one of the most obvious indicators of fight outcomes because strength is related to size; in intraspecific contests, the larger animal tends to dominate [26]–[30]. In interspecific contests (*e.g.*, a native species against an invader in the presence of niche overlap), the success of an invasive species in competition with a native species can be difficult to explain mainly based on differences in body size. A species-specific asymmetry in aggressiveness, for instance, might be sufficient to overcome an asymmetry in body size. Aggressiveness is a trait that can vary between individuals and therefore could contribute to RHP. Unlike other metrics of RHP (e.g. body size), however, ‘aggressiveness’ cannot be measured independently of agonistic encounters. In a recent study, for example, repeated observations of the same individuals in multiple agonistic encounters revealed aggressiveness to be both an RHP trait and a personality trait [31]. Thus, even a larger-bodied pearl cichlid might be less aggressive than a Nile tilapia and not have an advantage in a fight and would not be likely to gain access to a contested resource. This hypothesis agrees with a recent discussion that in intraspecific contest between green swordtails (*Xiphophorus helleri*), aggressiveness is determined only in part by the relative size (RHP) of an opponent towards which agonistic acts are directed, but also because it is a trait of individual personality [31]. In this context, as personality can modulate the aggressiveness of individuals, we postulate that aggressiveness might also be involved in interspecific contests; that is, some species would be more aggressive than others, irrespective of their body size. In this study, we compared dyadic interspecific fights staged between individual Nile tilapia and pearl cichlids differing in body size. The outcomes of these fights were examined to determine whether interspecific differences in aggressiveness might overcome the body-size effect. Our findings potentially identify a behavioural mechanism that could be linked to the competitive advantage of an invasive species against a native species. We tested this hypothesis using an invasive African cichlid, the Nile tilapia (*Oreochromis niloticus*), against a native Brazilian cichlid, the pearl cichlid (*Geophagus brasiliensis*).

## Materials and Methods

### Animal welfare statement

This research agrees with the Ethical Principles in Animal Research adopted by the National Council for the Control of Animal Experimentation - Brazil (CONCEA - Conselho Nacional de Controle de Experimentação Animal - Brazil) and was approved by the Ethical Committee for Animal Research from the Instituto de Biociências/UNESP (CEUA - Comissão de Ética no Uso de Animais), protocol 331.

### Animals and holding conditions

The pearl cichlid, *Geophagus brasiliensis* (Quoy & Gaimard 1824), were obtained from a lagoon system at Jardim Britânia, in a rural area of São Paulo city (23°25′46.34″S, 46°47′19.47″W), while the Nile tilapia, *Oreochromis niloticus* (L.), from a lagoon system at the aquacultural facility of CAUNESP (Centre of Aquaculture of São Paulo State University, Jaboticabal city, São Paulo state, Brazil). The mean standard length of the Nile tilapia specimens was 7.6±1.2 cm, and the standard length (the length of the caudal fin is excluded) of the pearl cichlid specimens was 7.7±1.4 cm. Sexually immature fishes were used because, in nature, competition among juveniles appears to be more relevant during competitive exclusion [14]. In this juvenile age, sex is not easily distinguishable and thus was not determined. The fishes were housed in separate stock tanks, therefore, communication between the individuals of the two study species was prevented prior to the beginning of the experiment. During this period, the individuals of each species were housed in a 120-L glass tank (70×35×50 cm; 1 fish/1.5 L) with a water temperature of 25±1°C, continuous aeration and mechanical and biological filtration, a natural photoperiod and natural indirect illumination. The levels of ammonia and nitrite were <0.5 ppm and <0.05 ppm, respectively. The animals were fed daily with commercial dry pellets (32% protein, Presence; Evialis do Brasil Nutricão Animal, Paulínia, SP, Brasil). Leftover food was removed periodically, and 60% of water was changed (dechlorinated water) at least once a week or when necessary (tendency of ammonia to increase).

### Experimental protocol and procedures

Our basic approach was to evaluate the frequency of attacks and dominance relations in pairs of fish consisting of a Nile tilapia and a pearl cichlid of specified body sizes. The fish were removed from the holding tanks, transferred to individual 20-L glass tanks (40×20×25 cm) and kept in isolation for 5 days to eliminate the effects of prior social experience [32]. The feeding regime and the water quality were identical to those used in the holding tanks. On the sixth day, a fish of each species was gently placed into the same neutral arena (40×20×25 cm) at the same time, and the agonistic interactions of the fish were video-recorded for 30 min. Six independent experimental pairings conditions, with n = 8 replicates each, were used to investigate the effects of differences in body size between the members of a pair. Each trial was conducted using the basic procedures outlined above. The size pairings used in the study involved pearl cichlids 30% or 10% smaller than the Nile tilapia, similar in size to the Nile tilapia, and Nile tilapia 10%, 30% or 50% smaller than the pearl cichlids. The standard body lengths used are given in Table 1.

**Table 1:** Standard body length of pearl cichlid and Nile tilapia.

Size parings	Species mean (±SD) standard length (cm)	
	Pearl cichlid (PC)	Nile tilapia (NT)
PC<NT (30%)	6.6±0.2	9.4±0.3
PC<NT (10%)	7.0±0.3	7.8±0.3
PC = NT	7.3±0.3	7.3±0.3
NT<PC (10%)	7.9±0.6	7.0±0.6
NT<PC (30%)	9.3±0.4	6.5±0.3
NT<PC (50%)	10.8±0.5	5.7±0.2

### Behavioural analyses

We quantified the aggressive interactions occurring in the videotaped experiments using previously published ethograms for the Nile tilapia [33] and the pearl cichlid [20]. We quantified the directed attacks by counting the numbers of biting on anterior (head), tail fin, median or ventral area; lateral fighting (a sudden slap between fish bodies) with fish oriented with the head in the same direction or in opposite directions; chasing; and mouth wrestling. The initiator of an attack was identified by observing who approached the opponent and directed the attack; the loser was the fish that left the place of attack or retreat. We identified the social status (dominant or subordinate) of each individual by calculating the dominance index, defined as the number of attacks directed by a fish divided by the total number of interactions exhibited by the pair [34].

### Statistical Analyses

A Kolmogorov–Smirnov test showed that the data were not normally distributed. A Bartlett's test revealed that the data were not homoscedastic, even after transformation. Because analysis of variance (ANOVA) is robust to variance heterogeneity in large balanced designs, the raw data were used in the model, but we used a conservative level of significance (*P*<0.01, see Underwood [35]). We used repeated-measures one-way ANOVA to analyse aggressive interactions (attacks) and to analyse the dominance index. In order to determine the effects of size asymmetry (between group factor; see Table 1 for levels of this category) and fish species (repeated measure; pearl cichlid or Nile tilapia) on the number of attacks and the dominance ranking, we used repeated measures one-way ANOVA (adapted from Briffa and Elwood [36]). The ANOVA was followed by a post hoc Student-Newman-Keuls test for multiple comparisons of the means.

## Results

**Figure 1:**
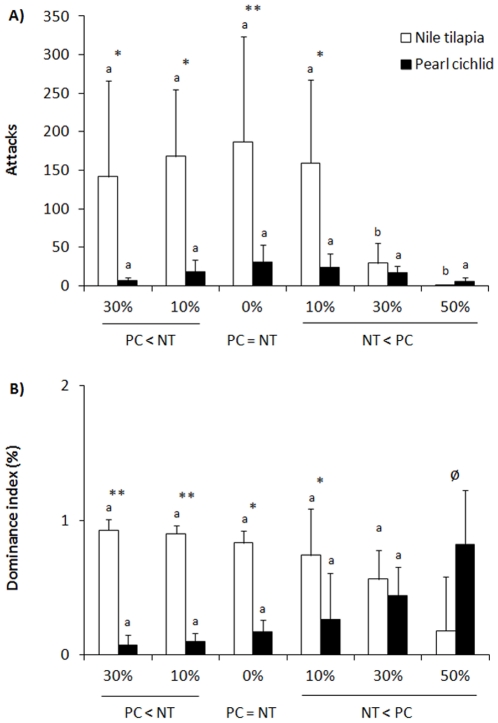
Aggressive interactions and dominance in fights staged between pearl cichlids and Nile tilapia. Unfamiliar fish were paired interspecifically in a neutral arena subsequent to 5 days of isolation, and aggressive behaviour was observed for 30 min. The fish differed in body size. The mean values (±SD; A - attack frequency; B – dominance index) that do not share the same letter are statistically different among fish of different body sizes for each species (*P*<0.01; one-way ANOVA with repeated measures followed by a Student-Newman-Keuls test). * *P*<0.01, ** *P*<0.001 denote that mean (±SD) values between species within the same condition are statistically different (one-way ANOVA with repeated measures followed by a Student-Newman-Keuls test). ø indicates that these values were not included in the statistical analyses (see the text for details).

A significant treatment x species interaction was found for the attack frequency (F_(5,42)_ = 4.729, P = 0.0016, Figure 1A) and for the dominance index (F_(4,35)_ = 4.659, P = 0.0040, Figure 1B). The frequency of attacks by the Nile tilapia was greater when the pearl cichlid were 30% smaller than the Nile tilapia, 10% smaller, similar in size or when Nile tilapia was 10% smaller than the pearl cichlids. In these pairs, the mean value of the number of directed attacks by the Nile tilapia was approximately 150. The rates of attacks by the tilapia decreased significantly when the Nile tilapias were 30% or 50% smaller than the pearl cichlids (less than 35 attacks). The rates of attack by the pearl cichlids did not vary with the body size of the tilapia, and the frequency of these attacks varied between 5 and 35.

The Nile tilapias were clearly more aggressive than the pearl cichlid, and the dominance index reflected these differences in aggression. The Nile tilapia was the dominant fish in the pair when pearl cichlid were 30% or 10% smaller than the Nile tilapia, similar in size, or Nile tilapias was 10% smaller than the pearl cichlid. When the Nile tilapia was 30% smaller than the pearl cichlid, no difference in the dominance index was observed; moreover, when the Nile tilapia was 50% smaller than the pearl cichlid, the attack frequency was substantially reduced. Thus, dominance index for these pairs was not compared with the dominance index for the other treatments. The pairs with this composition exhibited no more than 16 interactions and some pairs had fewer than 6 interactions. In this case, a fish could have a maximum dominance index (1 or 100%) by attacking only once if the other fish did not attack at all. Moreover, two pairs did not fight. These findings reinforce the high aggressiveness of the Nile tilapia and highlight the clear tendency of the Nile tilapia to become the dominant fish. Interestingly, body size did not have a strong influence on the outcomes of the fights or the determination of dominance.

## Discussion

This study demonstrates the marked success in fighting and the dominance of the invasive Nile tilapia over the native pearl cichlid (Brazil). This success occurred despite body-size differences and provides evidence of a behavioural mechanism for the extinction or drastic reduction of a native species by the Nile tilapia. The results of this study also indicate that ecological problems are possible in places where the Nile tilapia is grown in aquaculture, where accidental introductions of this species are likely to occur (e.g., see García et al. [37], in Argentina).

The Nile tilapia and the pearl cichlid are aggressive fish species [18]–[21] and, in nature, the former appears to demonstrate a competitive advantage over the pearl cichlid. Evidence for this advantage is suggested by the association between the occurrence of Nile tilapia and the decline of pearl cichlid populations, a phenomenon intensified by habitat degradation [14]. Body size (RHP) commonly modulates aggressive encounters among individuals of the same species and influences the outcome of an aggressive dispute, where the largest individual will generally dominate [26]–[30]. In the context of the interspecific aggressive interactions of the present study, Nile tilapia is generally more aggressive and pearl cichlid must be substantially larger to mitigate the aggression advantage of this invader species. Thus, the effects of body size (RHP) on the outcome of the fights or dominance in an interspecific context seem to figure as a secondary trait that does not occur in the way predicted for intraspecific aggressive encounters. However, Wilson et al. [31] state that intraspecific aggression should be explained by a connection between contest (RHP - body size) and personality (aggressiveness) theories. In the context of Wilson et al. 's conclusion, our conclusion is in line with this predicted direction and extends the connections between these theories to the interespecific level because species aggressiveness and relative size of the contestants counterbalance to determine fight outcome.

The results of this study showed that the Nile tilapia and the pearl cichlid differed in aggressiveness and that this effect prevailed over the body-size effect. The pearl cichlid was at a disadvantage in fights against the tilapia. When the Nile tilapias were larger, similar in size or even 10% smaller than the pearl cichlids, they were highly aggressive towards the pearl cichlids and dominated the encounter. When the Nile tilapia were 30% or 50% smaller than the pearl cichlid, the pearl cichlids should have had a clear advantage during the fight because of their body size; however, they did not become the dominant fish in the pair, and they did not act as aggressively as the Nile tilapia. We conclude that the aggressiveness of the Nile tilapia surpassed the body-size effect in the contests between the tilapia and the pearl cichlids. Similar effects in encounters between the invasive mosquitofish (*Gambusia affinis*) and the chub (*Lotichthys phlegethontis*) in Walter Spring, Utah, USA have been reported [9] and in laboratory experiments between the invasive Nile tilapia and the native sunfish, *Lepomis miniatus* (estuaries of the Gulf of Mexico) [12]. These studies, however, evaluated other behavioural mechanisms.

In a competitive interaction, small Nile tilapias could easily exclude pearl cichlids of different sizes. Due to its aggressiveness, the Nile tilapia exhibited a tendency to win in the contest with the pearl cichlid, or it did not succumb easily, irrespective of the size of the pearl cichlid. Thus, the Nile tilapia had a tendency to dominate in the fights against the pearl cichlid in most of the aggressive encounters that we staged between the juveniles of these species. Dominance hierarchies produce unequal distributions of resources, and the dominant individuals are able to monopolise the resources [38]. For instance, in a laboratory study of intraspecific contests, the dominant Nile tilapia made a patch of food unavailable to the subordinate specimens [39]. In the context of the present study, it is possible to extrapolate the effects of this sort to interspecific interactions between the pearl cichlids and Nile tilapia and to suggest an explanation based on the long-term competitive exclusion that has been reported by Linde et al. [14].

Field observations of fish (serranid – e.g., groupers and seabass) species have shown that agonistic interactions occur even between individuals of different life-history stages [40]. These interactions segregate spatially individuals of different body sizes, thus avoiding food overlap and, consequently, competitive exclusion [40]. However, contests between the Nile tilapia and the pearl cichlid have been reported to involve niche overlap [14]. Thus, the competitive advantage shown by the Nile tilapia in this study potentially represents one of several possible mechanisms of the negative interactions caused by an invasive species. These negative effects may reduce the viability of native species populations and cause competitive exclusion.
